# Complement-independent pathogenic influences of anti-HMGCR + and anti-SRP + immune-mediated necrotizing myopathy autoantibodies on engineered muscle function

**DOI:** 10.1186/s13395-025-00400-7

**Published:** 2025-11-25

**Authors:** Heta Lad, Yekaterina Tiper, Ernest Myguel Esteban, Manish K. Singh, Alexandrine Mahoudeau, Zhuoye Xie, Berenice Tendrel, Yves Allenbach, Olivier Benveniste, Penney M. Gilbert

**Affiliations:** 1 Institute of Biomedical Engineering, University of Toronto, Toronto, ON M5S3G9 Canada; 2 Donnelly Centre, University of Toronto, Toronto, ON M5S3E1 Canada; 3 Sorbonne University, INSERM, Center of Research in Myology, UMRS 974, Paris, 75013 France; 4 Department of Clinical Immunology and Internal Medicine, Sorbonne University, APHP, Pitié Salpêtrière Hospital, Paris, 75013 France; 5 Department of Cell and Systems Biology, University of Toronto, Toronto, ON M5S3G5 Canada

**Keywords:** Immune-mediated necrotizing myopathies, Autoantibodies, Disease modeling, Complement, Skeletal muscle atrophy, Stimulated contractile force

## Abstract

**Background:**

Immune-mediated necrotizing myopathy (IMNM) is a subgroup of idiopathic inflammatory myopathies associated with anti-signal recognition particle (SRP) or anti-3-hydroxy-3-methylglutaryl-CoA reductase (HMGCR) autoantibodies. The direct pathogenic effects of IMNM patient autoantibodies on skeletal muscle contractile force, independent of the downstream activation of the complement pathway, remain understudied.

**Methods:**

This study leverages a custom 3-D human skeletal muscle microtissue (hMMT) culture platform that encourages muscle cell contractile apparatus maturation and enables analysis of contractile function. Force generation competent hMMTs were treated with total immunoglobulins (IgGs) isolated from the plasma of IMNM patients with amplification of anti-SRP^+^ (*n* = 7) or anti-HMGCR^+^ (*n* = 7) autoantibodies and delivered in complement inactivated media for 4 days. hMMT function was then evaluated by quantifying the peak force and contraction kinetics in response to electrical field stimulation, followed by histological analysis of muscle cell gross morphology and sarcomere structure. To determine whether IgG from IMNM patients can enter muscle cells, 2-D myotube cultures were treated with donor total IgG delivered in complement replete media for 36 h, and then analyzed using immunostaining and confocal microscopy.

**Results:**

Exposure to total IgGs isolated from a subset of IMNM patients induced a decline in hMMT twitch and tetanus contractile force and were associated with sarcomere fragility and slowed muscle cell contraction and relaxation rates. Pathogenic influences on hMMT force generation were observed at a greater frequency in response to total IgGs isolated from IMNM patients with anti-HMGCR + autoantibodies. Substantial intracellular human IgG staining was observed in conditions where myotubes were treated with total IgGs from IMNM patients.

**Conclusions:**

This study demonstrates that total IgGs isolated from IMNM patients have the aberrant capacity to enter muscle cells in the absence of complement. Further, a subset of patient IgGs exert direct pathogenic influences on engineered muscle contractile function that are independent of the complement system. Together, these findings have important implications for the advancement of IMNM precision medicine therapies.

**Supplementary Information:**

The online version contains supplementary material available at 10.1186/s13395-025-00400-7.

## Background

Idiopathic inflammatory myopathies (IIM), also known as myositis, are a heterogenous family of autoimmune disorders where muscle weakness and myalgia are classical clinical manifestations developing gradually over a period of weeks to months, or even years, and frequently resulting in severe impairment [1–5]. Other organ systems may be additionally affected, and prompt life-threatening complications [1–5]. Advances in IIM studies have led to a classification of subtypes based on differences in histopathology or clinical manifestations, one of which is immune-mediated necrotizing myopathy (IMNM) [1, 2].

IMNM is the most severe and disabling form of myositis and accounts for 35% of all IIM cases [1, 3, 5]. This subgroup of IIMs is characterized by rapidly progressive muscle weakness and substantially elevated creatine kinase levels, often requiring early, aggressive immunotherapy [3–5]. To date, the presence of two autoantibodies (aAb) has been associated with IMNM; anti-signal recognition particle (SRP) or anti-3-hydroxy-3-methylglutaryl-CoA reductase (HMGCR) aAbs. Anti-SRP^+^ IMNM can be severe and progress rapidly, or it can progress slowly, mimicking limb-girdle muscular dystrophy [6, 7]. Anti-HMGCR^+^ IMNM is also severe and may occur during or after statin exposure [8].

Regarding immune-mediated necrotizing myopathy (IMNM) pathophysiology, where the diagnosis criteria were refined in an international workshop [9], we demonstrated that the specific antibody titer correlates with disease activity (muscle strength and creatine kinase levels) [10, 11] and that creatine kinase levels are an excellent biomarker of muscle fiber necrosis [12]. We have demonstrated the presence of sarcolemmal complement deposits, as well as the signs of classical complement pathway activation [12]. These findings suggest that muscle fiber necrosis is antibody- and complement-dependent. Accordingly, in muscle biopsies from patients with IMNM, no or few cytotoxic T cells and/or natural killer cells are observed [12]. With in vitro experiments in 2-D muscle cell cultures containing complement (i.e. animal serum), we have shown that specific autoantibodies are pathogenic [13]. Finally, experiments in mice indicated that the passive transfer of serum from IMNM patients with specific autoantibodies induces a myopathy that is complement-dependent [14] (see review article [5]).

Nonetheless, a phase II clinical trial testing a complement inhibitor, zilucoplan, did not improve the outcome measures in patients with IMNM [15]. This strategy has been shown to be effective in the prevention of disease occurrence in the mouse model [16]. However, its effectiveness in the treatment of induced disease is not as well-established. An alternative strategy that has shown more promise is the IgG depleting strategy in the mouse model using a neonatal FC receptor (FcRn) blocker (efgartigimod), which has been shown to work in a preventive and curative manner [17]. Consequently, ongoing therapeutic trials aim to reduce Ig half-life through FcRn receptor blockade (NCT05523167 and NCT05379634). Other antibody-depletion strategies have shown efficacy with daratumumab [18] and CD19-targeted CAR-T therapy [19, 20]. However, the hypothesis of pathogenic autoantibodies faces challenges due to the intracytoplasmic location of antibody targets and the limited success of anti-complement therapy [15]. Recent discoveries provide new supporting evidence, including the identification of genetic myopathy secondary to HMGCR loss of function [21] and the successful reproduction of pathogenic signatures in muscle culture models following intracellular introduction of IMNM antibodies [22, 23]. However, additional studies are needed to strengthen this emerging hypothesis.

To decouple pathogenic influences of IMNM patient autoantibodies on muscle form and function away from the impacts of the complement system, we implemented a three-dimensional (3-D) skeletal muscle microtissue system that allows the modeling of more physiologically and pathologically relevant processes ex vivo [24, 25]. Specifically, the skeletal muscle (Myo) microTissue Array device To Investigate forCe (MyoTACTIC) culture device enables the bulk production of 3-D human skeletal muscle microtissues (hMMTs) within a 96-well plate format wherein the deflection of flexible vertical posts enables in situ quantification of tissue strength and calcium transient behaviours [24, 26]. Leveraging this system, we treated hMMTs with total immunoglobulins (IgGs) purified from the plasma of anti-SRP^+^ and anti-HMGCR^+^ patients, and delivered within complement inactivated media, to investigate whether these aAbs induce a direct pathogenic effect on the force-generating capacity of hMMTs. Studies were also conducted to determine whether total IgGs from IMNM patients are able to enter muscle cells in the absence of the complement system.

## Methods

### Human samples and total IgG extraction

IMNM patient plasma was collected during plasmapheresis and used to extract total IgG. Pharmaceutical grade OCTAPLAS LG^®^ human plasma (Group A, Lot M726A9521) was purchased to extract healthy control IgG (OctaPharma AB). Before IgG extraction, samples were diluted (1:1) in BupH™ phosphate buffered saline (Cat. #28372, Thermo Scientific) and then centrifuged at 3200 g for 20 min to equilibrate pH and avoid column blockade. To extract total IgG from each sample, protein G spin columns were used by following the manufacturer instructions (Cat. #89957, Thermo Scientific). After extraction, total IgG eluate was concentrated using 30 kDa Amicon filters (Cat. #UFC9030, Millipore) and dialyzed once with Dulbecco’s Phosphate Buffered Saline (D-PBS; Cat. #14190-169, Gibco), and then concentrated again to obtain a stock concentration between 45 and 55 mg/ml. Total IgG concentration was quantified using NanoDrop 1000 Spectrophotometer before storage at −20 °C. Antigen recognition by patient antibodies was tested using a kit (for anti-SRP; Cat. #PMS12DIV-24, D-tek), or an automated machine (for anti-HMGCR; Cat. #701333, Inova Diagnostics) by following the supplier instructions in Dr. Charuel’s immunology laboratory (Pitié Salpêtrière University Hospital, Paris France). The total IgG concentration extracted for each sample and corresponding target recognition data is provided in Table 1 and the clinical features and characteristics of each plasma donor can be found in Table 2.

**Table 1:** Concentration and target recognition of total IgG samples extracted from plasma

Sample ID	Experiment ID	Pathology	[Total IgG] (mg/mL)	Target Recognition
SP1	Anti – SRP Patient 1	Anti – SRP	43.61	57U
SP2	Anti – SRP Patient 2	Anti – SRP	49.09	> 57U
SP3	Anti – SRP Patient 3	Anti – SRP	28.02	11U
SP4	Anti – SRP Patient 4	Anti – SRP	50.50	141U
SP5	Anti – SRP Patient 5	Anti – SRP	51.50	143U
SP6	Anti – SRP Patient 6	Anti – SRP	50.40	129U
SP7	Anti – SRP Patient 7	Anti – SRP	49.00	73U
HP1	Anti – HMGCR Patient 1	Anti – HMGCR	51.50	192.5 CU
HP2	Anti – HMGCR Patient 2	Anti – HMGCR	54.40	175.7 CU
HP3	Anti – HMGCR Patient 3	Anti – HMGCR	48.40	60.7 CU
HP4	Anti – HMGCR Patient 4	Anti – HMGCR	47.70	452 SGU
HP5	Anti – HMGCR Patient 5	Anti – HMGCR	50.40	> 550 SGU
HP6	Anti – HMGCR Patient 6	Anti – HMGCR	51.00	> 550 SGU
HP7	Anti – HMGCR Patient 7	Anti – HMGCR	48.30	> 550 SGU
Total Plasma Control	Healthy IgG	Negative Control	47.27	-

Recognition units: U (units), SGU (standard IgG b2GPI units), and CU (chemiluminescent units)

*SRP* Signal recognition particle, *HMGCR* 3-hydroxy-3-methylglutaryl-CoA reductase

**Table 2:** Clinical features and characteristics of patient plasma donors

Sample ID	Pathology	Sex	Age at Sampling	Treatment at sampling	CK Levels	MMT8	PGA
SP1	SRP	F	68	Corticosteroid, Methotrexate	3065	90	9
SP2	SRP	F	27	Solumedrol, Corticosteroid, IgIV	12,431	97	9
SP3	SRP	F	26	Corticosteroid, IgIV	7754	105	8
SP4	SRP	M	46	No treatment at sampling	6490	NA	NA
SP5	SRP	M	53	Corticosteroid, Methotrexate	2845	118	9
SP6	SRP	F	21	No treatment at sampling	7107	109	9
SP7	SRP	F	21	Corticosteroid, Methotrexate, Rituximab, IgIV	525	68	10
HP1	HMGCR	M	47	Zilucoplan, CellCept, IgIV, Cortancyl	1453	94	7
HP2	HMGCR	M	24	Cortancyl, Endoxan, IgIV	269	142	4
HP3	HMGCR	F	34	Corticosteroid	164	102	6
HP4	HMGCR	F	47	Corticosteroid, Cortancyl, IgIV, Methotrexate, Azathioprine	604	126	7
HP5	HMGCR	F	42	Cortancyl	7713	86	9
HP6	HMGCR	F	69	Corticosteroid, plasmapheresis	1854	118	8
HP7	HMGCR	F	65	Methotrexate	1265	118	8

*SRP* Signal recognition particle, *HMGCR* 3-hydroxy-3-methylglutaryl-CoA reductase, *CK* Creatine kinase, *MMT8 *Manual Muscle Testing 8, *PGA* Physician Global Assessment

### Myotactic culture platform fabrication

The polydimethylsiloxane (PDMS; Sylgard™ 184 silicone elastomer kit, Dow Corning) MyoTACTIC 96-well culture platform was used to generate human skeletal muscle micro-tissues (hMMTs). In this platform, each well contains an oval pool with two flexible PDMS micro-posts on either side of the pool. These posts provide uniaxial tension to induce hMMT self-organization while also enabling the non-invasive quantification of hMMT contractile function. The platform was fabricated exactly as we previously described [24, 26]. All features of the plate were cast in a single step from a reusable, negative polyurethane (PU; Smooth-Cast^®^ 310 liquid plastic; Smooth-On) mold. PDMS culture plates were prepared for muscle cell culture by first sonicating MyoTACTIC portions for 20 min in isopropanol (Cat. 8600), after which they were rinsed in ddH_2_O and set to evaporate in a curing oven for 15 min. MyoTACTIC portions were autoclaved for final sterilization before use.

### Human immortalized and primary myoblast cell line maintenance

The AB1167 (healthy) human-immortalized myoblast cell line used for the differentiation of 2-D myotubes and in the fabrication of hMMTs for this study was established at the Myoline platform of the Institut de Myologie (Paris, France) [27]. The human primary myoblast line used for the differentiation of 2-D myotubes was obtained from Cook MyoSite Inc. (SK-1111-P01358-19 F). It was derived using a proprietary method from the vastus lateralis muscle of a 19-year-old Caucasian female with a body mass index of 24 kg/m^2^ and no known medical conditions. The immortalized myoblast line was expanded, as previously described [25, 26], to produce the requisite number of cells for 2-D myotube differentiation and hMMT seeding. The primary myoblast line was expanded, as previously described [28, 29], to produce the requisite number of cells for 2-D myotube differentiation. A summary of all media and solutions used for cell line expansion can be found in Table 3.

**Table 3:** Composition of media and solutions for immortalized and primary myoblast maintenance, and 2D myotube differentiation

Media/Solution	Composition
Immortalized myoblast growth media	Skeletal Muscle Cell Growth Medium kit (Cat. #C-23160, Promo-cell), 15% FBS^1^ (Cat. #12483020, Gibco), 1% P/S^2^ (Cat. #15140122, Gibco)
Primary myoblast growth media	Ham’s F-10 nutrient mix (Cat. #318-050-CL, Wisent Bioproducts), 20% FBS, 5 ng/mL FGF2^3^ (Cat. #11343625, ImmunoTools), 1% P/S
Wash medium	DMEM^4^ (Cat. #11995065, Gibco), 5–10% FBS, 1% P/S
Freezing Media	90% FBS and 10% DMSO^5^
Immortalized 2D myotube after seeding growth media	Skeletal Muscle Cell Basal Medium (Cat. #C-23260, Promo-Cell), 20% FBS, 1% P/S
Primary 2D myotube after seeding growth media	Ham’s F-10 nutrient mix, 20% FBS, 1% P/S
2D myotube differentiation medium (primary & immortalized)	DMEM, 2% HS^6^ (with and without heat inactivation; Cat. #16050114, Gibco), 10 µg/mL bovine insulin (Cat. #I6634, Sigma), 1% P/S

^1^Fetal bovine serum (FBS)

^2^Penicillin-Streptomycin (P/S)

^3^Fibroblast growth factor 2 (FGF2)

^4^Dulbecco’s Modified Eagle’s Medium (DMEM)

^5^Dimethyl sulfoxide (DMSO)

^6^Horse serum (HS)

### hMMT seeding and total IgG treatment

3-D hMMTs were generated in the MyoTACTIC platform from AB1167 (healthy) immortalized cells exactly as were previously described [25, 26]. Following seeding, constructs were incubated in hMMT after seeding growth media (Day − 2). Two days later, the culture media was exchanged for differentiation media (DM; day 0) with half media changes carried out every other day until day 8 of differentiation. On day 8, the standard DM containing horse serum (HS; Cat. #16050114, Gibco) was removed and replaced with DM containing either HS, heat-inactivated (HI) HS, HI HS with cerivastatin (75 nM in ddH_2_O; Cat. #SML0005, Sigma), or HI HS with 1 mg/ml of healthy, anti-SRP, or anti-HMGCR total IgGs. On day 10, half of the media was refreshed with the respective treatment delivered at a 2x concentration, and end-point analyses were performed on day 12 of differentiation. Total IgG from seven patients producing anti-SRP autoantibodies and seven patients producing anti-HMGCR autoantibodies served as biological replicates (Tables 1 and 2). Three technical replicates were assessed per treatment group except the study reported in Supplementary Fig. 1 wherein two technical replicates were used for force analysis. A summary of all media and solutions used for hMMT seeding and culture can be found in Table 4.

**Table 4:** Composition of media and solutions for AB1167 hMMT production

Media/Solution	Composition
Fibrinogen stock solution	10 mg/mL fibrinogen (Cat. #F8630, Sigma) in 0.9% (wt/v) NaCl solution in water
Hydrogel mixture	DMEM^1^ (40% v/v; Cat. #11995065, Gibco), 4 mg/mL fibrinogen (40% v/v), Geltrex™ (20% v/v; Cat. #A1413202, Gibco)
hMMT after seeding growth media	Skeletal Muscle Cell Basal Medium (Cat. #C-23260, Promo-Cell), 20% FBS^2^ (Cat. #12483020, Gibco), 1.5 mg/mL ACA^3^ (3% v/v; Cat. #A2504, Sigma), 1% P/S^4^ (Cat. #15140122, Gibco)
hMMT differentiation medium	DMEM, 2% HS^5^ (with and without heat inactivation; Cat. #16050114, Gibco), 2 mg/mL ACA (4% v/v), 10 µg/mL human recombinant insulin (Cat# 91077 C, Sigma), 1% P/S

^1^Dulbecco’s Modified Eagle’s Medium (DMEM)

^2^Fetal bovine serum (FBS)

^3^6-Aminocaproic acid (ACA)

^4^Penicillin-Streptomycin (P/S)

^5^Horse serum (HS)

### 2-D myotube differentiation and total IgG treatment

2-D myotubes were differentiated on plastic 96-well tissue culture plates (Cat. #83.3924, Sarstedt) and glass-bottom 96-well PhenoPlates (Cat. #6055302, Revvity), using both immortalized and primary cells. Myoblasts were plated on Geltrex™ (Cat. #A1413202, Gibco) coated wells at ~ 30,000 cells/cm^2^. Following plating, cells were incubated in 2D myotube after seeding growth media. After one day, the culture media was exchanged for DM (day 0) and a half media change was carried out on day 2 of differentiation. On day 3, the standard DM containing horse serum was removed and replaced with DM containing HI HS alone or HI HS with 0.3 mg/ml of healthy, anti-SRP, or anti-HMGCR total IgGs. The experiment was terminated after 36 h of treatment on day 5 of differentiation for end point analyses. Total IgG from seven patients producing anti-SRP autoantibodies and seven patients producing anti-HMGCR autoantibodies served as biological replicates (Tables 1 and 2). Two wells per treatment group were assessed as technical replicates for the plastic plate and one well per treatment group was assessed for the glass bottom plate. A summary of all media and solutions used for 2-D myotube culture can be found in Table 3.

### Electrical field stimulation of hMMTs

Electrical field stimulation (EFS) was performed on day 12 of differentiation as we previously described [26]. A systematic optimization protocol determined that 0.5 Hz, 5 V, and 80 ms EFS parameters elicit peak force from AB1167 hMMTs and thus were used as the EFS conditions in this study [28]. Constructs were stimulated at room temperature (RT) immediately after removal from the incubator. Post movements in recorded videos were analyzed to measure the force and kinetics of hMMT contraction using a custom script. This custom, semi-automated script measures post displacement in recorded videos and generates a time course of post locations for the manual analysis of hMMT contraction kinetics. This study implemented an updated version of the script allowing for the simultaneous, semi-automated analysis of hMMT post displacement and kinetics (2.6 and 2.7). The script can be found here [https://github.com/gilbertlabcode/myoTACTIC].

### Evaluation of contractile force and contraction kinetics

Constructs were stimulated for 6–7 contractions at 0.5 Hz, 5 V, and 80 ms, parameters we have shown to elicit immortalized hMMT peak twitch in prior studies [28]. Following 30 s of rest, they were stimulated for 6–7 contractions at 60 Hz, 5 V, and 5 ms to elicit peak tetanic force. The first and second contractions generated by hMMTs were often irregular in comparison to subsequent contractions, and, thus, were excluded from analysis. Three hMMTs from a single MyoTACTIC portion served as replicates. For 0.5 Hz stimulus trains, we report the peak twitch force and the kinetics of the twitch contractions. Time-to-peak tension (TPT), half-relaxation time (1/2 RT), contraction rate, relaxation rate, duration-at-peak, and full-width at half max have been included. For stimulation at 60 Hz, we report the maximum absolute force. The absolute force generated during the second tetanic contraction was used to report peak force.

### Immunohistochemistry and image acquisition

At day 12 of differentiation, control and treated hMMTs were fixed in paraformaldehyde (Cat. #A11313, Alfa Aesar) for morphological analysis. To visualize myotubes within hMMTs, fixed constructs were immunostained for sarcomeric α-actinin (SAA), phalloidin to visualize f-actin, and Hoechst counterstained to observe nuclei. Confocal images were acquired at 40x magnification using the Fluoview-10 imaging software and an IX83 inverted confocal microscope (Olympus). The complete protocol for hMMT staining and imaging has been described [25]. A summary of all solutions and antibodies used to stain hMMTs can be found in Table 5.

**Table 5:** Solutions and antibodies used for hMMT immunohistochemical staining

Solutions	Composition	
Fixing solution	4% PFA^1^ in D-PBS^2^ (Cat. #311–415-CL, Wisent Bioproducts)	
Permeabilization and blocking solution	10% GS^3^ (Cat. #16210072, Gibco), 0.3% Triton X-100 (Cat. #TRX777, BioShop) in D-PBS	
Primary antibody solution	Mouse anti-SAA^4^ antibody (1:800; Cat. #A7811, Sigma) diluted in permeabilization and blocking solution	
Secondary antibody solution	Goat anti-mouse IgG conjugated with Alexa-Fluor™ 488 (1:500; Cat. #A-11001, Invitrogen), Phalloidin conjugated with Alexa Fluor™ 568 (1:400; Cat. #A12380, Invitrogen), and Hoechst 33,342 (1:1000; Cat. #H3570, Invitrogen), diluted in permeabilization and blocking solution	

^1^Paraformaldehyde (PFA)

^2^Dulbecco’s phosphate-buffered saline (D-PBS)

^3^Goat serum (GS)

^4^Sarcomeric α-actinin (SAA)

Control and treated 2-D myotubes were fixed in paraformaldehyde at day 5 of differentiation. To visualize 2-D myotubes, fixed samples were immunostained for sarcomeric α-actinin (SAA) and Hoechst counterstained to observe nuclei. A fluorescently tagged anti-human IgG antibody was included during secondary antibody staining to assess for the presence of intracellular human IgG. The complete protocol for 2-D myotube staining has been described [30]. A summary of all solutions and antibodies used to stain 2-D myotubes can be found in Table 6. Confocal images of samples cultured on glass bottom plates were acquired with an ~ 2 μm step size with each selected myotube imaged from top to bottom. The same acquisition parameters were applied to all samples when collecting images for this study and were selected based on an absence of signal in the secondary antibody control sample. A minimum of 25 regions of interest (ROI; 60 × 60 pixels) from z-projections were collected per treatment condition. Z-projection ROIs within the myotube region were selected (i.e. SAA+) taking care to avoid areas with non-specific signal caused by debris and the nucleus was avoided.

**Table 6:** Solutions and antibodies used for 2D immunohistochemical staining

Solutions	Composition	
Fixing solution	4% PFA^1^ in D-PBS^2^ (Cat. #311–415-CL, Wisent Bioproducts)	
Permeabilization solution	0.1% Triton X-100 (Cat. #TRX777, BioShop), 1% BSA^3^ (Cat. #ALB005, BioShop) in D-PBS	
Blocking solution	10% GS^4^ (Cat. #16210072, Gibco) in D-PBS	
Primary antibody solution	Mouse anti-SAA^5^ antibody (1:800; Cat. #A7811, Sigma) diluted in 1% GS in D-PBS	
Wash solution	0.025% Tween-20 (Cat. #TWN510, BioShop) in D-PBS	
Secondary antibody solution	Goat anti-mouse IgG conjugated with Alexa-Fluor™ 488 (1:500; Cat. #A-11001, Invitrogen) with goat anti-human IgG conjugated with Alexa-Fluor™ 555 (1:500; Cat. # A-21433, Invitrogen), and Hoechst 33,342 (1:1000; Cat. #H3570, Invitrogen), diluted in 1% GS in D-PBS	

^1^Paraformaldehyde (PFA)

^2^Dulbecco’s phosphate-buffered saline (D-PBS)

^3^Bovine serum albumin (BSA)

^4^Goat serum (GS)

^5^Sarcomeric α-actinin (SAA)

### Myotube width and coefficient of variance analysis

Confocal images of the SAA channel were utilized to quantify hMMT average myotube width and coefficient of variance. Analysis of flattened image stacks was facilitated by the Fiji Software (ImageJ, NIH), and calculation of these morphometrics was performed in complete accordance with our previously reported methods [25].

### Nuclear fusion index and SAA coverage analysis

Confocal images of the SAA and Hoechst channels were utilized to quantify hMMT nuclear fusion index and SAA coverage. All analysis and calculations were performed in complete accordance with our previously reported methods [25].

### Automated analysis of Z-line architecture

The Z-line architecture of hMMT sarcomeres was assessed using a computational structural assay developed in MATLAB by Morris et al. [31]. In short, this program called ZlineDetection extracts a binary skeleton of z-lines from immunostained images of SAA and f-actin which it then uses for the quantification of z-line architecture parameters. For this analysis, hMMT SAA and f-actin confocal images were processed, and the program was run using the same modifications to author-recommended settings as we previously reported [25]. The mean continuous Z-line length, mean number of Z-lines per image, mean sarcomere length, and Orientation Order Parameter (OOP) were evaluated.

### Intracellular human IgG quantification

Human IgG intensity measurements were collected from unmanipulated 2D myotube raw image ROIs using the ImageJ ROI manager. The mean intensity of each ROI was represented on graphs. 3D renderings of selected confocal stacks was generated using the following ImageJ plugins: 3D Objects Counter and 3D suite. After subtracting background using a Gaussian filter, an object image was created using the 3D Objects Counter plugin. The object image was then added to the 3D suite plugin and visualized using the 3D viewer plugin. Surfaces were smoothened through 7 iterations. A 360-degree rotation movie was recorded for representation.

### Statistical analysis

**Fig. 1:**
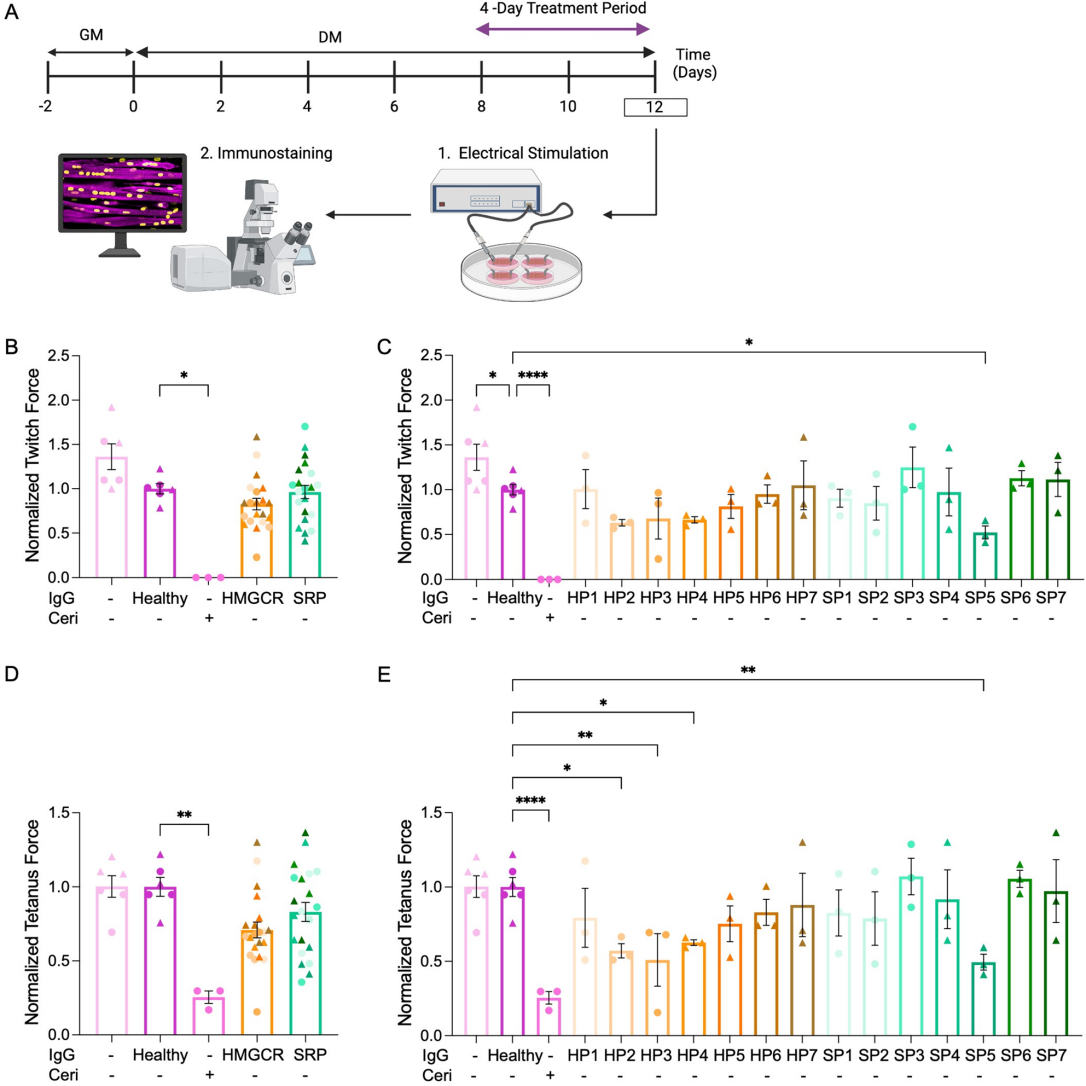
A subset of IMNM patient autoantibodies reduce hMMT contractile force.(A) A schematic representation of the hMMT culture timeline and endpoint analyses. hMMTs are cultured in after seeding growth media (GM) and then switched to differentiation media (DM) for the next 12 days of culture. On day 8 of differentiation, the horse serum containing DM is removed and replaced by DM prepared using heat-inactivated horse serum with or without patient total IgGs. On day 12 of differentiation, endpoint analysis is conducted. (B-E) Dot plots of hMMT mean twitch (B-C) or tetanic (D-E) force with patient groups clustered (B,D) or separated (C,E). N = 7 patients for anti-HMGCR+ IgG treatments (anti-HMGCR Patient 1 [HP1], anti-HMGCR Patient 2 [HP2], and anti-HMGCR Patient 3 [HP3] and so on) and N=7 patients for anti-SRP+ IgG treatments (anti-SRP Patient 1 [SP1], anti-SRP Patient 2 [SP2], and anti-SRP Patient 3 [SP3] and so on). Cerivastatin (Ceri, 75 nM) treatment served as a positive control and no added IgG (-) or Healthy IgG treatment served as negative controls. n = 3 hMMTs per treatment condition. Samples were tested across N=2 independent experiments which are distinguished in graphs by circle and triangle datapoints. All values are reported as means SEM; * p ≤ 0.05, ** p < 0.01, and *** p < 0.001

Statistical analysis was performed using GraphPad Prism 10.0. All values are expressed as mean ± standard error of the mean (SEM). Significance was defined as *p* ≤ 0.05. Table 7 lists the statistical test used for each figure, as well as technical replicate information for each biological replicate. For EFS experiments, Rout’s outlier test, where the maximum desired false discovery rate (Q) was set to Q = 5%, was used to identify outliers. This resulted in the removal of two data points in the anti-HMGCR group within the “Tetanus (uN)” dataset in Fig. 1D. Statistical tests were then run following the removal of these outliers. Contractile apparatus maturation can vary across independent replicates, necessitating a titration of the Cerivastatin dosage needed to induce atrophy. In one experiment the dose failed to induce atrophy. As such, these datapoints (*n* = 3 tissues) were excluded from the downstream analyses. A spreadsheet containing all raw data has been provided [see Additional File 1].

**Table 7:** Technical and biological replicate breakdown and statistical analysis tests

Figure	Technical (hMMTs) and independent biological replicates	Images per technical replicate (hMMT)	*n* to calculate statistics/error bars	Statistical Test
SI 1B	*n* = 3 across *N* = 1	5 images	*n* = 3	Student’s t-test (unpaired, two-tailed)
SI 1 C - D	*n* = 2 across *N* = 1	-	*n* = 2	Student’s t-test (unpaired, two-tailed)
SI 2	Healthy IgG, *n* = 6 for *N* = 2 HMGCR and SRP, *n* = 3 for each of *N* = 7 biological replicates analyzed over *N* = 2 independent experiments	-	SI 2 A, *n* = 6 SI 2B, *n* = 3 SI 2 C, *n* = 3 SI 2D, HI HS and Healthy IgG, *n* = 6. HP1-7, SP1-7,*n* = 3 SI 2E, *n* = 3 SI 2 F, *n* = 3	Simple linear regression
SI 3	*n* = 1, *N* = 1	2–3 images	Healthy IgG, *n* = 72 2nd CTRL, *n* = 58 HP1, *n* = 63 HP2, *n* = 25 HP3, *n* = 35 HP4, *n* = 37 HP5, *n* = 31 HP6, *n* = 29 HP7, *n* = 30 SP1, *n* = 73 SP2, *n* = 80 SP3, *n* = 81 SP4, *n* = 79 SP5, *n* = 84 SP6, *n* = 91 SP7, *n* = 109	Ordinary one-way ANOVA; Dunnett’s multiple comparisons test, comparing all means to the mean of the ctrl (Healthy IgG)
1B/D	IgG -/Ceri -, *n* = 6 from *N* = 2 Healthy IgG, *n* = 6 from *N* = 2 Ceri +, *n* = 3 HMGCR and SRP, *n* = 3 for each of *N* = 7 biological replicates analyzed over *N* = 2 independent experiments	-	IgG -, Ceri -, *n* = 6 Ceri +, *n* = 3 Healthy IgG, *n* = 6 HMGCR, *n* = 21 SRP, *n* = 21	Non-parametric one-way ANOVA; Kruskal Wallis multiple comparisons test comparing all means to the mean of the ctrl (Healthy IgG)
1 C/E	IgG -/Ceri -, *n* = 6 from *N* = 2 Healthy IgG, *n* = 6 from *N* = 2 Ceri +, *n* = 3 HMGCR and SRP, *n* = 3 for each of *N* = 7 biological replicates analyzed over *N* = 2 independent experiments	-	IgG -/Ceri -, *n* = 6 Ceri +, *n* = 3 Healthy IgG, *n* = 6 HMGCR, *n* = 3 for each of the *N* = 7 SRP, *n* = 3 for each of the *N* = 7	One-way ANOVA; Uncorrected Fisher’s LSD test
2 A – F	IgG -/Ceri -, *n* = 6 from *N* = 2 Healthy IgG, *n* = 6 from *N* = 2 Ceri +, *n* = 3 DCE, *n* = 3 for each of *N* = 4 biological replicates analyzed over *N* = 2 independent experiments NCE, *n* = 3 for each of *N* = 10 biological replicates analyzed over *N* = 2 independent experiments	-	IgG -/Ceri -, *n* = 6 Ceri +, *n* = 3 Healthy IgG, *n* = 6 DCE, *n* = 12 NCE, *n* = 30	Non-parametric one-way ANOVA; Kruskal Wallis multiple comparisons test comparing all means to the mean of the ctrl (Healthy IgG)
3B	IgG -/Ceri -, *n* = 6 from *N* = 2 Healthy IgG, *n* = 6 from *N* = 2 Ceri +, *n* = 3 DCE, n = ~ 3 for each of *N* = 4 biological replicates analyzed over *N* = 2 independent experiments NCE, n = ~ 3 for each of *N* = 10 biological replicates analyzed over *N* = 2 independent experiments	3 images	IgG -/Ceri -, *n* = 6 Ceri +, *n* = 3 Healthy IgG, *n* = 3 HMGCR, *n* = 11 SRP, *n* = 29	Non-parametric one-way ANOVA; Kruskal Wallis multiple comparisons test comparing all means to the mean of the ctrl (Healthy IgG)
3 C	IgG -/Ceri -, *n* = 6 from *N* = 2 Healthy IgG, *n* = 6 from *N* = 2 Ceri +, *n* = 3 DCE, n = ~ 3 for each of *N* = 4 biological replicates analyzed over *N* = 2 independent experiments NCE, n = ~ 3 for each of *N* = 10 biological replicates analyzed over *N* = 2 independent experiments	4–5 images	IgG -/Ceri -, *n* = 6 Ceri +, *n* = 3 Healthy IgG, *n* = 3 HMGCR, *n* = 11 SRP, *n* = 29	Non-parametric one-way ANOVA; Kruskal Wallis multiple comparisons test comparing all means to the mean of the ctrl (Healthy IgG)
3D	IgG -/Ceri -, *n* = 259 myotubes Healthy IgG, *n* = 276 myotubes Ceri +, *n* = 138 myotubes DCE, *n* = 578 myotubes across *N* = 4 biological replicates analyzed over *N* = 2 independent experiments NCE, *n* = 1528 myotubes across *N* = 10 biological replicates analyzed over *N* = 2 independent experiments	4–5 images	IgG -/Ceri -, *n* = 259 myotubes Ceri +, *n* = 138 myotubes Healthy IgG, *n* = 276 myotubes DCE, *n* = 578 myotubes NCE, *n* = 1528 myotubes	Non-parametric one-way ANOVA; Kruskal Wallis multiple comparisons test comparing all means to the mean of the ctrl (Healthy IgG)
3E	IgG -/Ceri -, *n* = 6 from *N* = 2 Healthy IgG, *n* = 6 from *N* = 2 Ceri +, *n* = 3 Anti-HP1, *n* = 3 Anti-HP2, *n* = 3 Anti-HP3, *n* = 3 Anti-HP4, *n* = 3 Anti-HP5, *n* = 3 Anti-HP6, *n* = 3 Anti-HP7, *n* = 2 Anti-SP1, *n* = 3 Anti-SP2, *n* = 3 Anti-SP3, *n* = 3 Anti-SP4, *n* = 3 Anti-SP5, *n* = 2 Anti-SP6, *n* = 3 Anti-SP7, *n* = 3 ^ For all size bins *n* = 3 (compiled from 4–5 images per n)	4–5 images	IgG -/Ceri -, *n* = 6 Ceri +, *n* = 3 Healthy IgG, *n* = 6 Anti-HP1, *n* = 3 Anti-HP2, *n* = 3 Anti-HP3, *n* = 3 Anti-HP4, *n* = 3 Anti-HP5, *n* = 3 Anti-HP6, *n* = 3 Anti-HP7, *n* = 2 Anti-SP1, *n* = 3 Anti-SP2, *n* = 3 Anti-SP3, *n* = 3 Anti-SP4, *n* = 3 Anti-SP5, *n* = 2 Anti-SP6, *n* = 3 Anti-SP7, *n* = 3	-
3 F	IgG -/Ceri -, *n* = 6 from *N* = 2 Healthy IgG, *n* = 6 from *N* = 2 Ceri +, *n* = 3 DCE, n = ~ 3 for each of *N* = 4 biological replicates analyzed over *N* = 2 independent experiments NCE, n = ~ 3 for each of *N* = 10 biological replicates analyzed over *N* = 2 independent experiments	4–5 images	IgG -/Ceri -, *n* = 6 Ceri +, *n* = 3 Healthy IgG, *n* = 3 HMGCR, *n* = 11 SRP, *n* = 29	Non-parametric one-way ANOVA; Kruskal Wallis multiple comparisons test comparing all means to the mean of the ctrl (Healthy IgG)
4B-E	IgG -/Ceri -, *n* = 6 from *N* = 2 Healthy IgG, *n* = 6 from *N* = 2 Ceri +, *n* = 3 DCE, n = ~ 3 for each of *N* = 4 biological replicates analyzed over *N* = 2 independent experiments NCE, n = ~ 3 for each of *N* = 10 biological replicates analyzed over *N* = 2 independent experiments	4–5 images	IgG -/Ceri -, *n* = 6 Ceri +, *n* = 3 Healthy IgG, *n* = 3 HMGCR, *n* = 11 SRP, *n* = 29	Non-parametric one-way ANOVA; Kruskal Wallis multiple comparisons test comparing all means to the mean of the ctrl (Healthy IgG)
5B	*N* = 1, *n* = 1	2–3 images	Healthy IgG, *n* = 98 2nd CTRL, *n* = 45 HP1, *n* = 71 HP2, *n* = 70 HP3, *n* = 70 HP4, *n* = 76 HP5, *n* = 75 HP6, *n* = 74 HP7, *n* = 112 SP1, *n* = 100 SP2, *n* = 80 SP3, *n* = 75 SP4, *n* = 82 SP5, *n* = 88 SP6, *n* = 87 SP7, *n* = 89	Ordinary one-way ANOVA; Dunnett’s multiple comparisons test, comparing all means to the mean of the ctrl (Healthy IgG)

## Results

### Complement-depleted serum does not negatively impact hMMT structure or function

To understand how patient autoantibodies directly affect muscle health, the pre-existing complement proteins found within the horse serum (HS) used to generate hMMTs were removed by heat inactivation. We then sought to determine whether the heat-inactivated horse serum (HI HS) impacted the human skeletal muscle microtissues (hMMTs) by conducting a comparative phenotypic and functional analysis. hMMTs were differentiated to day 8 in HS (field standard), a time-point when myonuclear accretion has plateaued and tissues are capable of contracting in response to electrical or chemical stimuli. From day 8 to day 12 of differentiation, hMMTs were cultured with HS (standard), or HI HS, and then immunostained to visualize sarcomeric α–actinin and nuclei (Fig. 1A). Gross morphology and quantitative morphometric analysis revealed that the myotube diameter within the HS and HI HS media conditions was comparable (Figure S1A-B). In a separate experiment, microtissues were subjected to electrical field stimulation (EFS) during which videos were recorded to capture the deflection of the flexible, vertical rubber posts that each tissue was constructed across (Fig. 1A). These studies revealed that the force-generating capacity of hMMTs differentiated with or without HI HS was comparable (Figure S1C-D). Thus, HI HS was used in media formulations for all subsequent studies.

### Total IgGs from a subset of IMNM patients induce a decline in hMMT tetanic force

Next, we investigated the direct effect of anti-SRP and anti-HMGCR aAbs on hMMT contractile force in the complement depleted experimental setting (Fig. 1). Total IgGs were extracted from plasmapheresis collected from 7 anti-SRP + and 7 anti-HMGCR + patients (Tables 1 and 2). On day 8 of differentiation, the striated, contractile myotubes within the hMMTs were introduced to HI HS DM containing 1 mg/ml total IgGs. At the day 12 culture endpoint, hMMTs were subjected to EFS to evaluate tissue function, and then these “stress-tested” (i.e. 6–7 twitch contractions followed by 6–7 tetanus contractions) hMMTs were immediately fixed and immunostained for morphometric analyses (Fig. 1A). During low-frequency EFS, quantification of average twitch force did not uncover statistically significant impacts of tissue contractile force in the presence of anti-HMGCR + or anti-SRP + aAbs at the population level (Fig. 1B). However, analyzing the twitch force data at the single patient resolution uncovered trended decreases in twitch force across tissues treated with total IgGs from each of three anti-HMGCR + IMNM patients (HP2 *p* = 0.08, HP3 *p* = 0.12, HP4 *p* = 0.10), as well as a statistically significant decline in twitch force induced by total IgGs from one anti-SRP + IMNM patient (Fig. 1C). A trended decline in absolute contractile force in response to high-frequency EFS was observed amongst the cohort of tissues treated with anti-HMGCR + aAbs, but not anti-SRP + aAbs (Fig. 1D; HMGCR *p* = 0.08). By visualizing the tetanic contractile force data of hMMTs at the individual HMGCR^+^ and SRP^+^ IMNM patient level, the same 3 HMGCR^+^ patient samples and the single SRP^+^ patient sample induced a significant decline in hMMT tetanic contractile force (Fig. 1E). From this, we concluded that a subset of IMNM patients produce aAbs that induce a pathogenic decline in hMMT force production that is independent of the complement system. Further, aAb-induced direct pathogenic influences were observed at a higher frequency amongst anti-HMGCR + IMNM patients, as compared to anti-SRP + IMNM patients.

### Directly pathogenic total IgGs from IMNM patients diminish hMMT relaxation rate

**Fig. 2:**
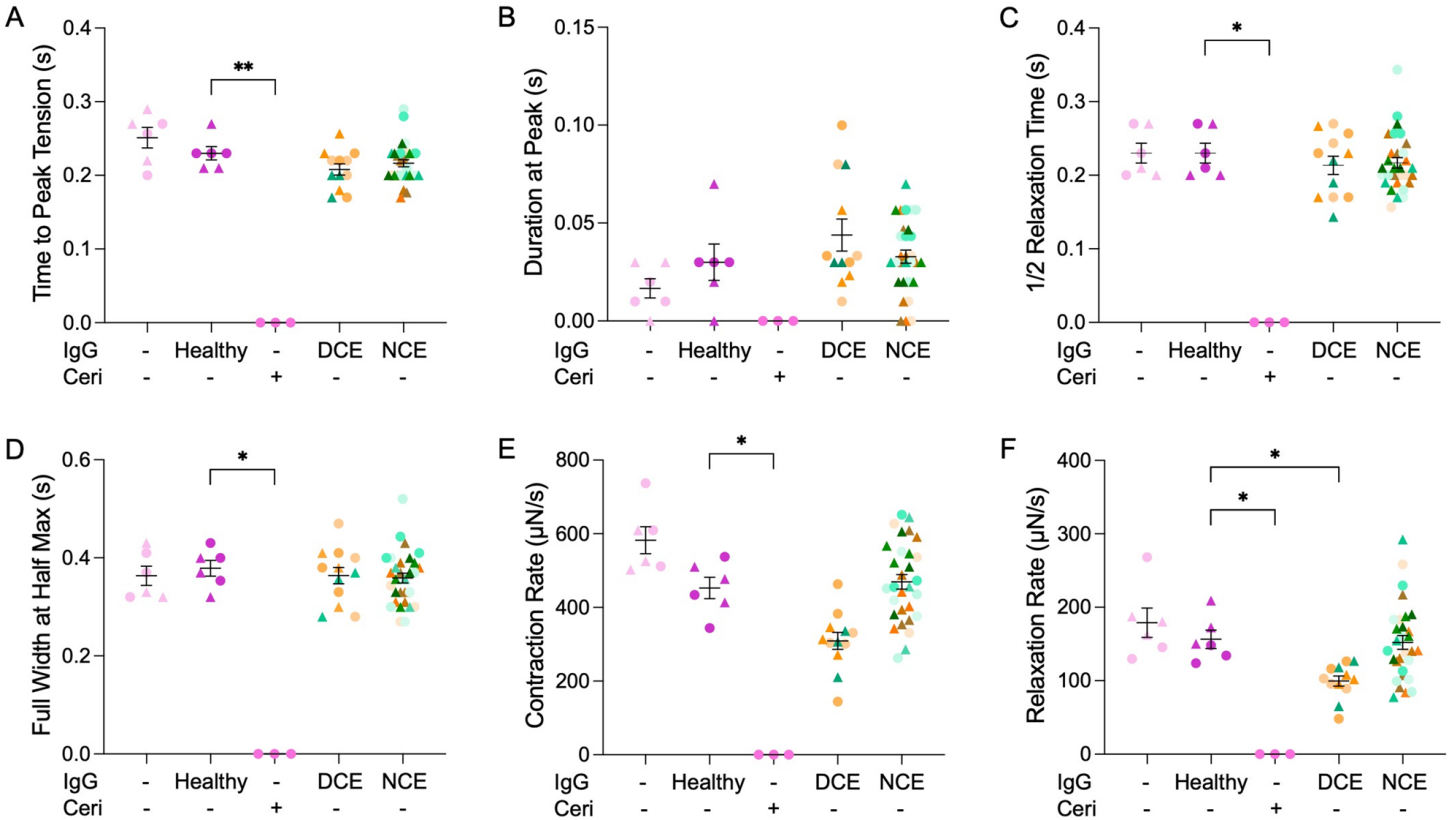
Directly pathogenic IMNM autoantibodies diminish hMMT contraction and relaxation rates. Dot plot quantification of time-to-peak tension (**A**), duration-at-peak (**B**), half-relaxation (**C**), full-width at half-maximum (**D**), contraction rate (**E**), and relaxation rate (**F**). *N* = 7 for anti-HMGCR + and *N* = 7 anti-SRP + IgG treatments. Cerivastatin (Ceri, 75 nM) treatment served as a positive control and no added IgG (-) or Healthy IgG treatment served as negative controls. *n* = 3 hMMTs per treatment condition. Samples were tested across *N* = 2 independent experiments which are distinguished in graphs by circle and triangle datapoints. Data for hMMTs treated with total IgGs are binned according to whether they induced a direct contractile effect (DCE; HP2-4 and SP5) or no contractile effect (NCE; HP1, 5–7 and SP1-4, 6, 7) on hMMT force production as indicated by the data in Fig. 1. All values are reported as means $\:\pm\:$ SEM; * *p* ≤ 0.05, ** *p* < 0.01, *** *p* < 0.001, and **** *p* < 0.0001

To hone in on the aspect of hMMT contractile function that was impacted by exposure to total IgGs isolated from IMNM patients where a direct contractile effect on hMMT force capacity was observed (DCE; HP2-4 and SP5), we evaluated contraction kinetics in response to low-frequency EFS. Quantification of time-to-peak tension (Fig. 2A), duration-at-peak (Fig. 2B), half-relaxation time (Fig. 2C), and full-width at half-maximum (Fig. 2D) revealed no significant impairments following exposure to the IMNM patient aAbs that induced no contractile effect (NCE; HP1, 5–7 and SP1-4, 6-7) or a direct contractile effect (DCE; HP2-4 and SP5) on hMMT force production (Fig. 1), relative to the Healthy IgG control. However, IMNM aAbs that induced a direct contractile effect on hMMT force caused a trended reduction in the rate of hMMT contraction (Fig. 2E), and a significant decline in the rate of relaxation (Fig. 2F). These deficits (Fig. 2E-F) in contraction kinetics during low stimulation EFS suggest disruptions in the calcium handling system, with a more dominant impact on calcium uptake into the sarcoplasmic reticulum.

### Loss of hMMT contractile force is not associated with a decline in myotube width

**Fig. 3:**
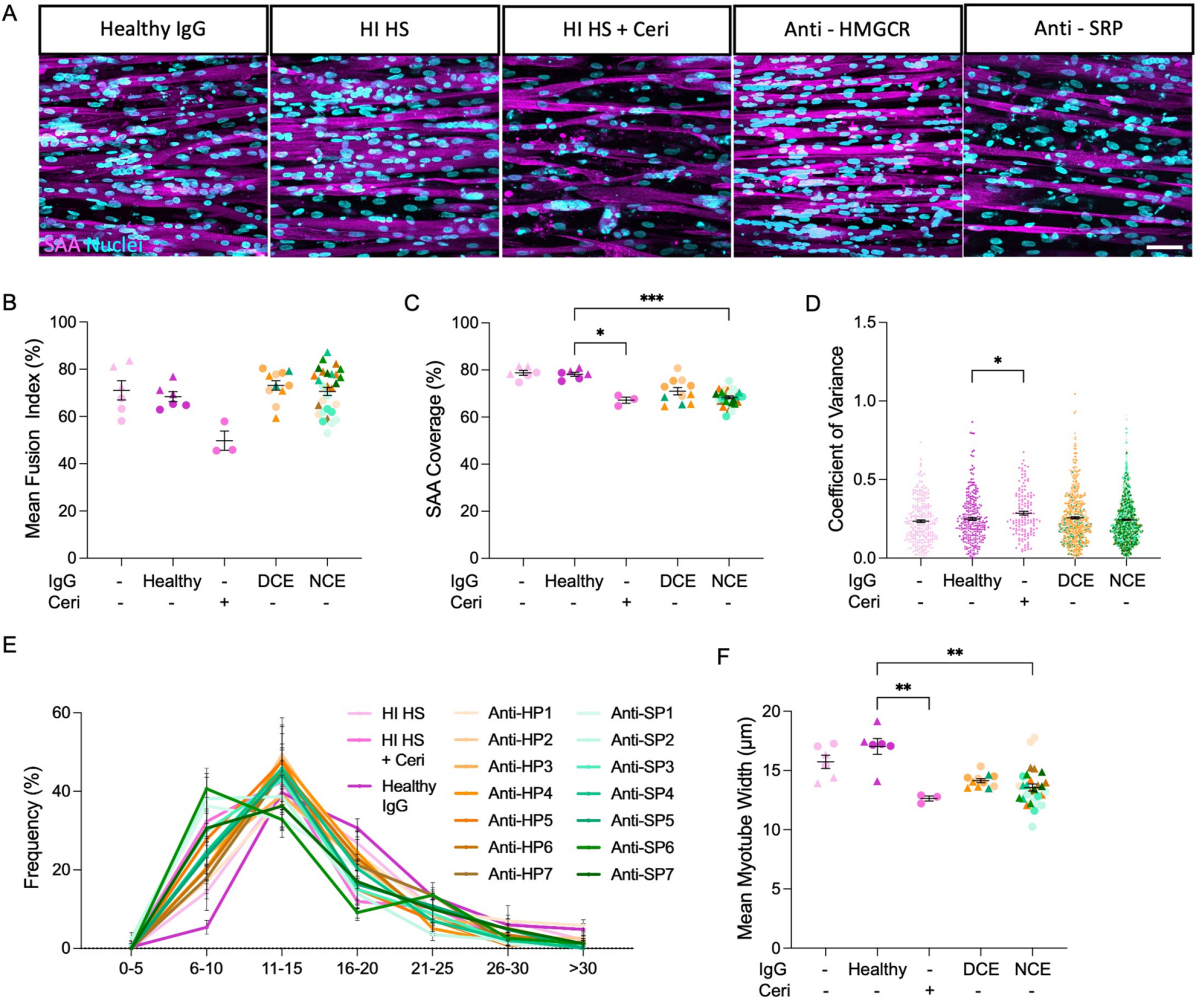
IMNM autoantibodies induce hMMT myotube atrophy. **A** Representative 40x confocal images of myotubes formed in hMMTs within each condition immunostained for sarcomeric α-actinin (SAA, magenta) and counterstained with Hoechst 33,342 (cyan). Scale bar = 50 μm. **B** Dot plot of hMMT nuclear fusion index for individual hMMTs. **C** Dot plot of mean SAA positive coverage within flattened confocal stack images for individual hMMTs. **D** Dot plot of myotube width variation as quantified by the coefficient of variance for single myotubes. *n* = 146 for (+) and (-) healthy IgG, *n* = 138 for Ceri, *n* = 524 for anti-HMGCR, and *n* = 550 for anti-SRP. **E** Histogram illustrating myotube diameter frequency across treatment conditions. **F** Dot plot showing average myotube diameter for individual hMMTs. *N* = 7 patients for anti-HMGCR + and *N* = 7 for anti-SRP + IgG treatments. Cerivastatin (Ceri, 75 nM) treatment served as a positive control and no added IgG (-) or Healthy IgG treatment served as negative controls. *n* = 3 hMMTs per treatment condition. Samples were tested across *N* = 2 independent experiments which are distinguished in graphs by circle and triangle datapoints. Data for hMMTs treated with total IgGs are binned according to whether they induced a direct contractile effect (DCE; HP2-4 and SP5) or no contractile effect (NCE; HP1, 5–7 and SP1-4, 6, 7) on hMMT force production as indicated by the data in Fig. 1. All values are reported as means $\:\pm\:$ SEM; * *p* < 0.05,** *p* < 0.01, *** *p* < 0.001, and **** *p* < 0.0001

We next examined whether treatment with total IgGs from patients with anti-HMGCR + and/or anti-SRP + autoantibodies led to alterations in myotube morphology. Sarcomeric α–actinin and Hoechst 33342 immunostaining at day 12 of differentiation revealed the formation of multinucleated, striated, and aligned myotubes in all groups (Fig. 3A) as well as comparable nuclear fusion indices (Fig. 3B). A reduction in the proportion of each tissue encompassed by sarcomeric α–actinin^+^ structures was observed for treatment groups exerting no contractile effect (NCE; HP1, 5–7 and SP1-4, 6, 7) on hMMT force production (Fig. 3C). While variance in the diameter along the length of myotubes was unaltered compared to the Healthy IgG control (Fig. 3D; co-efficient of variance), a decrease in myotube width for these treatment groups (Figs. 3E-F) accounts for the gross loss of muscle tissue observed in Fig. 3C. By contrast, treatment groups where a direct contractile effect (DCE; HP2-4 and SP5) on hMMT force production had occurred (Fig. 1) showed no gross loss of muscle tissue relative to the healthy IgG control, while trended impacts on individual myotube morphometrics were revealed (Figs. 3D-F). Thus, the vast majority of IMNM patients produce aAbs that cause engineered muscle cells to atrophy, without an impact on force production. However, the loss of hMMT strength that follows exposure to total IgGs from a subset of IMNM patients is not explained by myotube atrophy alone. Indeed, the relationship between force and myotube width is lost following hMMT treatment with total IgGs from IMNM patients (Figure S2A-C).

### Pathogenic total IgG treated hMMTs characterized by decreased sarcomere integrity

**Fig. 4:**
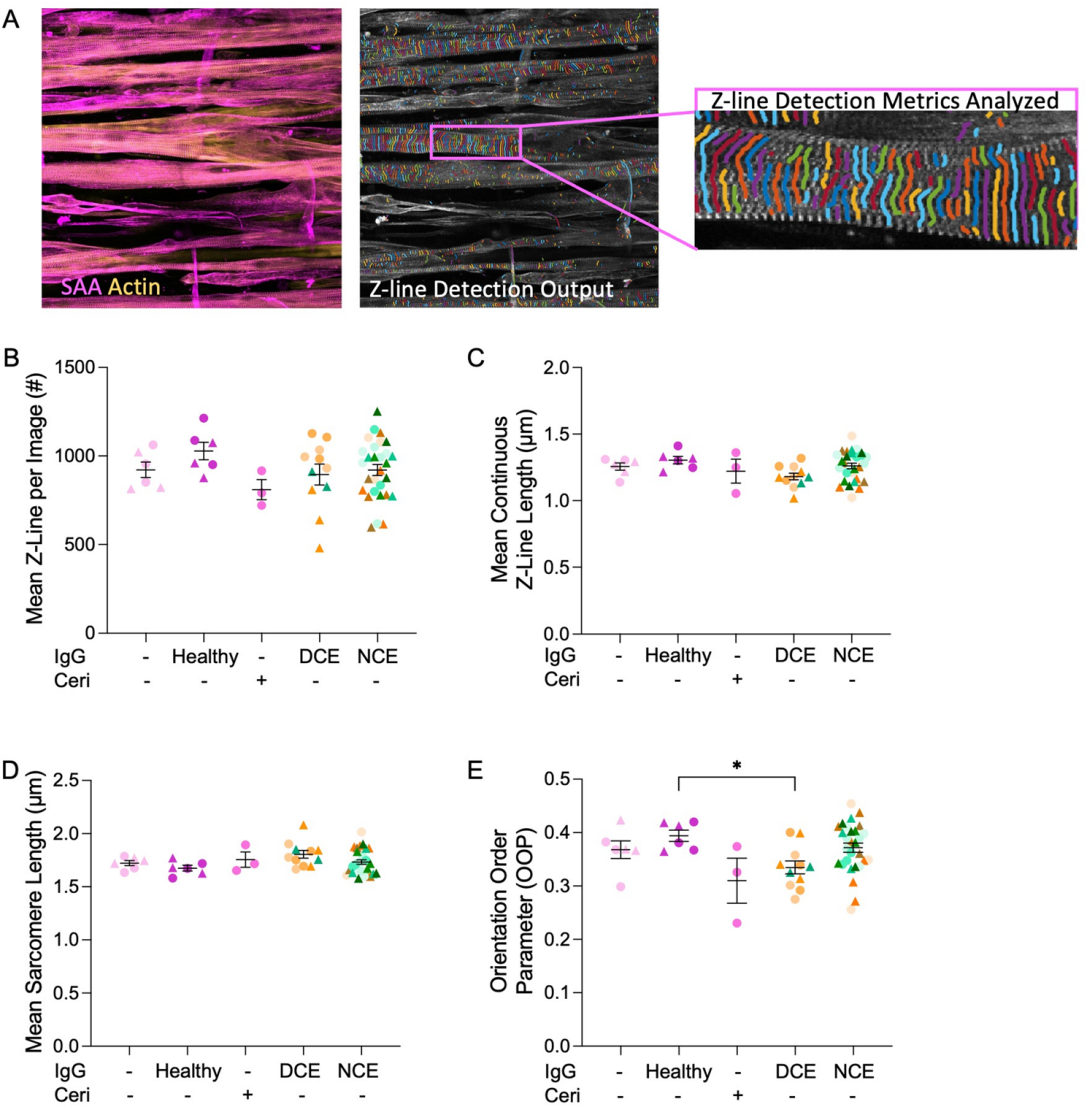
Sarcomere structures are disrupted after mechanical load. **A** Left panel shows a representative 40x confocal image of hMMT myotubes post electrical field stimulation immunostained for sarcomeric α-actinin (SAA, magenta) and f-actin (actin, yellow) which corresponds to the input for the ZlineDetection program. Scale bar = 50 μm. Middle panel shows a corresponding output from the ZLineDetection program with a further zoom in (right panel) to illustrate the retention of striations following a mechanical load under control conditions. **B**-**E** Dot plots of mean (**B**) Z-lines per image, (**C**) continuous Z-line length, (**D**) sarcomere length, and (**E**) orientation order parameter (OOP). *N* = 7 patients for anti-HMGCR + and *N* = 7 patients for anti-SRP + IgG treatments. Cerivastatin (Ceri, 75 nM) treatment served as a positive control and no added IgG (-) or Healthy IgG treatment served as negative controls. *n* = 3 hMMTs per treatment condition. Samples were tested across *N* = 2 independent experiments which are distinguished in graphs by circle and triangle datapoints. Data for hMMTs treated with total IgGs are binned according to whether they induced a direct contractile effect (DCE; HP2-4 and SP5) or no contractile effect (NCE; HP1, 5–7 and SP1-4, 6, 7) on hMMT force production as indicated by the data in Fig. 1. All values are reported as means $\:\pm\:$ SEM; * *p* ≤ 0.05

Following EFS, we immunostained the hMMTs to visualize sarcomeric α–actinin and f-actin (Fig. 4A, left panel) to analyze the stability of the sarcomere structures following a mechanical load “stress test” (i.e. 6–7 twitch contractions followed by 6–7 tetanus contractions) [25]. We implemented an automated analysis tool that enables assessment of sarcomere architecture from phenotypic data. Using this, we evaluated the mean number of z-lines per image, mean continuous z-line length, mean sarcomere length, and the sarcomere orientation order parameter (OOP) (Fig. 4A, middle and right panels). hMMTs arising from treatment groups exerting no contractile effect (NCE; HP1, 5–7 and SP1-4, 6, 7) on hMMT force production (Fig. 1) displayed no changes in sarcomere structures compared to the Healthy IgG control (Figs. 4B-E). Interestingly, hMMTs from treatment groups where a direct contractile effect (DCE; HP2-4 and SP5) on hMMT force production was observed (Fig. 1) showed a statistically significant decline in the OOP, a metric scored out of 1 (Fig. 4E). A trended decline in mean continuous z-line length (Fig. 4C; *p* = 0.08) was also uncovered by the analysis. Taken together, these data suggest that sarcomere development proceeds to a similar degree across all treatment conditions, but that sarcomere fragility in response to repeated contractile activity contributes to the loss of hMMT force production observed in response to treatment with total IgGs from a subset of IMNM patients. Indeed, a linear relationship exists between hMMT force production and sarcomere OOP underlying the relevance of this metric (Figure S2D-F).

### IMNM patient autoantibodies can enter muscle cells in culture

**Fig. 5:**
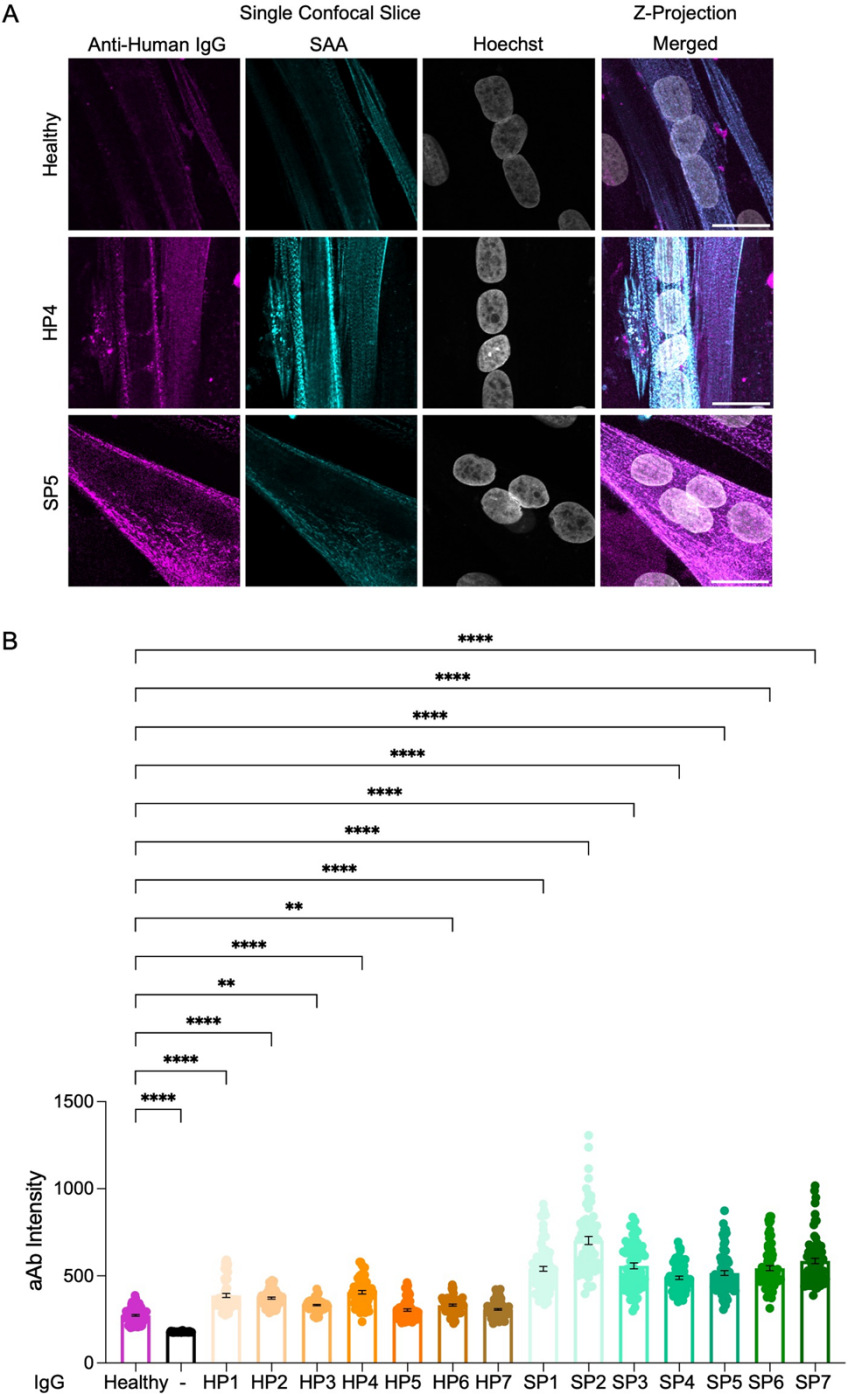
IMNM patient autoantibodies enter immortalized myoblast-derived myotubes in 2-D culture. **A** Representative z-projection (far right image) and single confocal slices (far left and middle images) of an immortalized myoblast-derived myotube in 2-D culture that was treated with total IgG from a healthy donor or an IMNM patient with HMGCR+ (HP4) or SRP+ (SP5) autoantibodies and then immunostained for anti-human IgG (magenta), sarcomeric α-actinin (SAA, cyan) and a Hoechst nuclear counterstain (gray). Scale bar = 20 μm. **B** Dot plot displaying mean human IgG immunostaining intensity quantified for individual regions of interest in immortalized myoblast-derived myotubes treated with total IgGs. *N* = 7 patients for anti-HMGCR + IgG treatments (anti-HMGCR Patient 1 [HP1], anti-HMGCR Patient 2 [HP2], and anti-HMGCR Patient 3 [HP3] and so on) and *N* = 7 patients for anti-SRP + IgG treatments (anti-SRP Patient 1 [SP1], anti-SRP Patient 2 [SP2], and anti-SRP Patient 3 [SP3] and so on). All values are reported as means $\:\pm\:$ SEM; ** *p* < 0.01 and **** *p* < 0.0001

Next, we investigated the possibility that IMNM patient autoantibodies may exert pathogenic effects by entering muscle cells via a complement-independent mechanism. To test this hypothesis, we produced myotubes in 2-D culture using human immortalized myoblasts (Fig. 5) or human primary myoblasts (Figure S3). On day 3 of differentiation, the standard media was replaced with HI HS DM alone or HI HS DM containing 0.3 mg/ml of total IgGs isolated from healthy donors, or the IMNM patients with anti-SRP+ (*n* = 7) or anti-HMGCR+ (*n* = 7) aAbs. After 36 h of treatment, the cultures were fixed and incubated with a fluorescently conjugated antibody recognizing human IgG alongside staining to visualize intracellular markers (sarcomeric a-actinin and Hoechst nuclear stain). In reviewing single confocal slices collected from each of the treatment groups (Figs. 5A and S3A), alongside 3D reconstruction movies of the confocal stack data (Additional Files 2–4), we found human IgG reactivity in immortalized and primary myotubes treated with each of the fourteen IMNM patient total IgG samples we tested. We then quantified the mean intensity of intracellular human IgG staining. Some intracellular human IgG was detected in myotubes treated with total IgGs from healthy donors (Fig. 5B and S3B), which is to be expected since the electrophysiologically immature sarcolemma of myotubes in 2D culture has a degree of porosity [32]. By comparison, all of the IMNM patient total IgG treated myotubes exhibited a mean intracellular human IgG signal that was well above the negative control (no IgG treatment) and statistically significantly higher than myotubes exposed to total IgG from the healthy donors (Fig. 5B and S3B). These data suggest that IMNM patient autoantibodies may be able to enter muscle cells in the absence of complement, but that pathogenic effects on sarcomere form and function are tied to mechanisms beyond access to the intracellular space.

## Discussion

Herein we deliver evidence for complement-independent, direct pathogenic effects of anti-HMGCR + and anti-SRP + IMNM patient aAbs on sarcomere stability and the contractile function of engineered human skeletal muscle. This work was enabled by an advanced skeletal muscle culture platform, which we leveraged to decouple the secondary immune influences of IMNM-associated aAbs on skeletal muscle function; allowing direct pathogenic influences to be uncovered. Our work suggests that only a subset of IMNM patients produce aAbs that induce a decline in sarcomere structure and muscle force production in the absence of complement influences, and we note that an enrichment for direct influences on contractile activity was observed in response to total IgG samples collected from IMNM patients with anti-HMGCR + aAbs. Furthermore, we present evidence showing that IMNM patient IgGs have the capacity to enter the intracellular space of multinucleated muscle cells. Consistent with our prior studies with complement-containing media [13], statistically significant and trended declines in myotube width were observed when hMMTs were treated with all IMNM patient total IgGs we tested. Collectively, these observations support a theory whereby aAbs are able to enter skeletal muscle cells and contribute to disease pathogenesis independent of complement system influences.

CAR-T cell therapy for autoimmune disorders is emerging as a breakthrough in the field [33]. Indeed, CAR-T cells targeting CD19 B cells [33] or B cell maturation antigen [34] have shown impressive results in treating idiopathic myositis patients, and anti-SRP IMNM patients more specifically, in the latter case. The success of targeting antibody-producing B cells for destruction emphasizes the critical need to understand the pathogenic role of Abs in IMNM to support a precision medicine approach to IMNM treatment and guide the next generation of therapeutic cell products. In the future it would be informative to conduct studies using anti-SRP and anti-HMGCR aAbs, isolated from the total IgG fraction, alongside antibody blocking studies, to understand whether these disease associated aAbs are indeed responsible for direct pathogenic effects, or whether additional modifiers present within the total IgG fraction drive or support pathogenic activities. Evaluating contractile strength after a period of removing IMNM total IgGs from the media has potential to understand whether the system can predict recovery of force following plasmapheresis in patients. Such findings would have important implications in the development of treatment plans and therapies to treat IMNM.

While our prior work has shown a strong relationship between specific antibody titer and disease activity (muscle strength and creatine kinase levels) in the clinic [10, 11], we observed no clear relationship between patient clinical data (Tables 1 and 2; sex, age, disease severity at time of sampling, target recognition strength, treatments) and our in vitro outcomes. This lack of predictive power may be explained by the short timeline of our in vitro treatments (4 days in 3-D and 36 h in 2-D). In the current study, hMMTs were treated with 0.3 mg/mL to 1 mg/mL of total IgGs, which is about 10-fold less than IgG concentration in human plasma, which may also explain discrepancies. However, it must be noted that it remains unclear what IgG concentration that muscle fibers are exposed to in vivo. Finally, it is possible that in vivo, other cell types residing in skeletal muscle modify disease progression and severity, justifying the implementation of a multicellular 3-D culture model to decouple these influences [35]. While attaining an in vitro assay that faithfully reflects patient clinical features is a lofty aspiration, exploring each of these avenues has potential to achieve the goal while also uncovering important insights into IMNM disease pathogenesis.

By contrast to healthy IgG control treated hMMTs, amongst our anti-SRP + and anti-HMGCR + aAb-treated hMMTs, we did not see a positive correlation between myotube width and contractile force in our in vitro studies (Figure S2A-C). While muscle size and strength are generally positively correlated based on clinical data, the relationship between the two can be complex and depend on various factors [36, 37]. Specifically, in IMNM, muscle size and strength can be independently influenced by the degree of inflammation, age of onset, the effectiveness or type of treatment, and individual variation such as comorbidities, to name a few factors. For instance, steroid therapy is considered the first line of therapy for general myositis. In spite of an increase in muscle strength after steroid therapy, researchers observed a significant loss of muscle mass even when there was no underlying disease, suggesting that the therapy itself decreases muscle volume [38]. While electrophysiological properties of myotube sarcolemma within our 3-D cultures closely match native human muscle cells [32], the contractile apparatus and calcium handling system remains underdeveloped in the absence of nerves [39]. This may form the basis of our ability to observe significant correlation between contractile force production and sarcomere orientation order parameter (Figure S2D-F), but also justifies expanding our functional metrics to include assessments of skeletal muscle excitation-contraction coupling [24, 25, 40–42].

## Conclusions

Together, our findings corroborate a complement-independent, direct role of anti-SRP and anti-HMGCR aAbs in IMNM pathogenesis, supporting a humoral-based disease mechanism that could be targeted in the treatment of IMNM. We offer compelling evidence in support of the theory that IMNM patient aAbs can enter muscle cells, independent of the complement cascade, where they then drive disease progression. Additionally, we show the value in studying the role of IMNM aAbs, ex vivo, in an advanced skeletal muscle culture system, as a strategy to deliver new insights into IMNM disease pathogenesis.

## Supplementary Information

Supplemental Fig. 1. Complement-depleted horse serum does not affect hMMT myotube width or force. (A) Representative 40x confocal images of myotubes formed in hMMTs cultured in the presence of horse serum for 8 days followed by 4 days in horse serum (HS; left) or heat-inactivated horse serum (HI HS; right) and then immunostained for sarcomeric α–actinin (SAA, magenta) and counterstained with Hoechst 33342 (cyan). Scale bar = 50 μm. (B) Dot plot showing average myotube diameter quantified for individual hMMTs. *n* = 3 hMMTs per condition. (C-D) Dot plots showing average (C) twitch and (D) tetanus contractile forces generated by electrical field stimulation of hMMTs. *n* = 2 hMMTs per condition. All values are reported as mean SEM, * *p* ≤ 0.05. Supplemental Fig. 2. hMMT strength correlates with sarcomere organization. The average myotube diameter (µm) and contractile force (µN) of each hMMT treated with (A) Healthy IgG or total IgG from (B) anti-HMGCR + or (C) anti-SRP + IMNM patients was plotted on the x and y axes, respectively. The mean contractile force (µN) of each hMMT treated with Healthy IgG or total IgG from anti-HMGCR + or anti-SRP + IMNM patients was plotted on the y-axes against mean (D) contraction rate, (E) relaxation rate, or (F) sarcomere orientation order parameter. Linear regressions were used to determine the goodness of fit with R^2^ and p-values reported. Supplemental Fig. 3. IMNM patient autoantibodies enter primary myoblast-derived myotubes in 2-D culture. (A) Representative z-projection (far right image) and single confocal slices (far left and middle images) of a primary myoblast-derived myotube in 2-D culture that was treated with total IgG from a healthy donor or an IMNM patient with HMGCR+ (HP4) or SRP+ (SP5) autoantibodies and then immunostained for anti-human IgG (magenta), sarcomeric α-actinin (SAA, cyan) and a Hoechst nuclear counterstain (gray). Scale bar = 20 μm. (B) Dot plot displaying mean human IgG immunostaining intensity quantified for individual regions of interest in primary myoblast-derived myotubes treated with total IgGs. *N* = 7 patients for anti-HMGCR + IgG treatments (anti-HMGCR Patient 1 [HP1], anti-HMGCR Patient 2 [HP2], and anti-HMGCR Patient 3 [HP3] and so on) and *N* = 7 patients for anti-SRP + IgG treatments (anti-SRP Patient 1 [SP1], anti-SRP Patient 2 [SP2], and anti-SRP Patient 3 [SP3] and so on). All values are reported as means SEM; ** *p* < 0.01 and **** *p* < 0.0001.Additional File 1. An Excel spreadsheet (.xls) with tabs corresponding to each Figure of the manuscript and containing the raw data corresponding to each subpanel. The file is available in the FigShare repository entitled Lad and Tiper et al IMNM Manuscript Additional File 1, doi: 10.6084/m9.figshare.29955449.Additional File 2. A 360-degree confocal stack rotation movie (.avi) of a representative 2-D immortalized myotube treated with total IgG isolated from a healthy control donor, immunostained to visualize human IgG (magenta), sarcomeric alpha-actinin (cyan), and nuclei (gray), imaged and then 3-D rendered, smoothened and recorded for presentation. The file is available in the FigShare repository entitled Lad and Tiper et al IMNM Manuscript Additional Files 2-4, doi: 10.6084/m9.figshare.29955449.Additional File 3. A 360-degree confocal stack rotation movie (.avi) of a representative 2-D immortalized myotube treated with total IgG isolated from an IMNM patient with HMGCR+ autoantibodies (HP4), immunostained to visualize human IgG (magenta), sarcomeric alpha-actinin (cyan), and nuclei (gray), imaged and then 3-D rendered, smoothened and recorded for presentation. The file is available in the FigShare repository entitled Lad and Tiper et al IMNM Manuscript Additional Files 2-4, doi: 10.6084/m9.figshare.29955449.Additional File 4. A 360-degree confocal stack rotation movie (.avi) of a representative 2-D immortalized myotube treated with total IgG isolated from an IMNM patient with SRP+ autoantibodies (SP5), immunostained to visualize human IgG (magenta), sarcomeric alpha-actinin (cyan), and nuclei (gray), imaged and then 3-D rendered, smoothened and recorded for presentation. The file is available in the FigShare repository entitled Lad and Tiper et al IMNM Manuscript Additional Files 2-4, doi: 10.6084/m9.figshare.29955449.

## Data Availability

The datasets generated and analyzed during the current study are available in the FigShare repository entitled Lad and Tiper et al. IMNM Manuscript Additional File 1, 10.6084/m9.figshare.29955449.

## Abbreviations

aAb: Autoantibodies
Ceri: Cerivastatin
DM: Differentiation media
D-PBS: Dulbecco’s Phosphate Buffered Saline.
EFS: Electrical field stimulation
GM: Growth media
HI: Heat-inactivated
HMGCR: 3-hydroxy-3-methylglutaryl-CoA reductase
HS: Horse serum
hMMT: Human skeletal muscle microtissue
IIM: Idiopathic inflammatory myopathies
IgGs: Immunoglobulins
IMNM: Immune-mediated necrotizing myopathy
MyoTACTIC: Skeletal Muscle (Myo) microTissue Array device To Investigate forCe
FcRn: Neonatal FC receptor
OOP: Orientation order parameter
PDMS: Polydimethylsiloxane
SAA: Sarcomeric α-actinin
SRP: Signal recognition particle
SEM: Standard error of the mean
