# Supplementary material for: Complement-independent pathogenic influences of anti-HMGCR + and anti-SRP + immune-mediated necrotizing myopathy autoantibodies on engineered muscle function
Source: Skelet Muscle. 2025 Nov 25;16:8. doi: 10.1186/s13395-025-00400-7 (PMC12882220; doi:10.1186/s13395-025-00400-7)
Supplement: Supplementary file 1 — Supplemental Fig. 1. Complement-depleted horse serum does not affect hMMT myotube width or force. (A) Representative 40x confocal images of myotubes formed in hMMTs cultured in the presence of horse serum for 8 days followed by 4 days in horse serum (HS; left) or heat-inactivated horse serum (HI HS; right) and then immunostained for sarcomeric α–actinin (SAA, magenta) and counterstained with Hoechst 33342 (cyan). Scale bar = 50 μm. (B) Dot plot showing average myotube diameter quantified for individual hMMTs. n = 3 hMMTs per condition. (C-D) Dot plots showing average (C) twitch and (D) tetanus contractile forces generated by electrical field stimulation of hMMTs. n = 2 hMMTs per condition. All values are reported as mean SEM, * p ≤ 0.05. Supplemental Fig. 2. hMMT strength correlates with sarcomere organization. The average myotube diameter (µm) and contractile force (µN) of each hMMT treated with (A) Healthy IgG or total IgG from (B) anti-HMGCR + or (C) anti-SRP + IMNM patients was plotted on the x and y axes, respectively. The mean contractile force (µN) of each hMMT treated with Healthy IgG or total IgG from anti-HMGCR + or anti-SRP + IMNM patients was plotted on the y-axes against mean (D) contraction rate, (E) relaxation rate, or (F) sarcomere orientation order parameter. Linear regressions were used to determine the goodness of fit with R2 and p-values reported. Supplemental Fig. 3. IMNM patient autoantibodies enter primary myoblast-derived myotubes in 2-D culture. (A) Representative z-projection (far right image) and single confocal slices (far left and middle images) of a primary myoblast-derived myotube in 2-D culture that was treated with total IgG from a healthy donor or an IMNM patient with HMGCR+ (HP4) or SRP+ (SP5) autoantibodies and then immunostained for anti-human IgG (magenta), sarcomeric α-actinin (SAA, cyan) and a Hoechst nuclear counterstain (gray). Scale bar = 20 μm. (B) Dot plot displaying mean human IgG immunostaining intensity quantified for [file 13395_2025_400_MOESM1_ESM.docx]

**Supplemental Figure 1. Complement-depleted horse serum does not affect hMMT myotube width or force.** (A) Representative 40x confocal images of myotubes formed in hMMTs cultured in the presence of horse serum for 8 days followed by 4 days in horse serum (HS; left) or heat-inactivated horse serum (HI HS; right) and then immunostained for sarcomeric α–actinin (SAA, magenta) and counterstained with Hoechst 33342 (cyan). Scale bar = 50 µm. (B) Dot plot showing average myotube diameter quantified for individual hMMTs. n = 3 hMMTs per condition. (C-D) Dot plots showing average (C) twitch and (D) tetanus contractile forces generated by electrical field stimulation of hMMTs. n = 2 hMMTs per condition. All values are reported as mean SEM, * p ≤ 0.05.

**Supplemental Figure 2. hMMT strength correlates with contraction kinetics and sarcomere organization, but not myotube width.** The average myotube diameter (µm) and contractile force (µN) of each hMMT treated with (A) Healthy IgG or total IgG from (B) anti-HMGCR+ or (C) anti-SRP+ IMNM patients was plotted on the x and y axes, respectively. The mean contractile force (µN) of each hMMT treated with Healthy IgG or total IgG from anti-HMGCR+ or anti-SRP+ IMNM patients was plotted on the y-axes against mean (D) contraction rate, (E) relaxation rate, or (F) sarcomere orientation order parameter. Linear regressions were used to determine the goodness of fit with R^2^ and p-values reported.

**Supplemental Figure 3. IMNM patient autoantibodies enter primary myoblast-derived myotubes in 2-D culture.** (A) Representative z-projection (far right image) and single confocal slices (far left and middle images) of a primary myoblast-derived myotube in 2-D culture that was treated with total IgG from a healthy donor or an IMNM patient with HMGCR+ (HP4) or SRP+ (SP5) autoantibodies and then immunostained for anti-human IgG (magenta), sarcomeric α-actinin (SAA, cyan) and a Hoechst nuclear counterstain (gray). Scale bar = 20 µm. (B) Dot plot displaying mean human IgG immunostaining intensity quantified for individual regions of interest in primary myoblast-derived myotubes treated with total IgGs. N = 7 patients for anti-HMGCR+ IgG treatments (anti-HMGCR Patient 1 [HP1], anti-HMGCR Patient 2 [HP2], and anti-HMGCR Patient 3 [HP3] and so on) and N=7 patients for anti-SRP+ IgG treatments (anti-SRP Patient 1 [SP1], anti-SRP Patient 2 [SP2], and anti-SRP Patient 3 [SP3] and so on). All values are reported as means $\pm$ SEM; ** p < 0.01 and **** p < 0.0001.
